# A fragmented neural network ensemble method and its application to image classification

**DOI:** 10.1038/s41598-024-52945-0

**Published:** 2024-01-27

**Authors:** Xu Zhang, Shuai Liu, Xueli Wang, Yumei Li

**Affiliations:** School of Mathematics and Statistics, Beijing Technology and Business University, Beijing, China

**Keywords:** Computer science, Information technology, Statistics

## Abstract

In recent years, deep neural networks have evolved rapidly in engineering technology, with models becoming larger and deeper. However, for most companies, developing large models is extremely costly and highly risky. Researchers usually focus on the performance of the model, neglecting its cost and accessibility. In fact, most regular business scenarios do not require high-level AI. A simple and inexpensive modeling method for fulfilling certain demands for practical applications of AI is needed. In this paper, a Fragmented neural network method is proposed. Inspired by the random forest algorithm, both the samples and features are randomly sampled on image data. Images are randomly split into smaller pieces. Weak neural networks are trained using these fragmented images, and many weak neural networks are then ensembled to build a strong neural network by voting. In this way, sufficient accuracy is achieved while reducing the complexity and data volume of each base learner, enabling mass production through parallel and distributed computing. By conducting experiments on the MNIST and CIFAR10 datasets, we build a model pool using FNN, CNN, DenseNet, and ResNet as the basic network structure. We find that the accuracy of the ensemble weak network is significantly higher than that of each base learner. Meanwhile, the accuracy of the ensemble network is highly dependent on the performance of each base learner. The accuracy of the ensemble network is comparable to or even exceeds that of the full model and has better robustness. Unlike other similar studies, we do not pursue SOTA models. Instead, we achieved results close to the full model with a smaller number of parameters and amount of data.

## Introduction

In recent years, deep neural networks have evolved rapidly in engineering applications. Model complexity is continuously increased, moving from the convolutional neural network (CNN) as the classic image recognition model and the recurrent neural network (RNN) as a sequence data processing model. Many specific model designs have been proposed for various scenarios, such as You Only Look Once (YOLO) for object detection^1^, Generative Adversarial Networks (GAN) for video restoration^2^, Bidirectional Encoder Representation from Transformers (BERT) for natural language processing^3^, and GPT-3^4^ created by Google for question-and-answer systems. These models represent the current trends of AI, which is massive, specialized and expensive. However, for most small and medium enterprises, the enormous computing power and labor costs are unaffordable. For example, the number of parameters of the GPT-3 mentioned above reached 17.5 billion. Moreover, in terms of business practices, such an investment is enormous and yields slow returns. Sometimes, generating direct benefits is difficult, and a high risk exists in R&D and productization. Therefore, in AI applications, we sometimes need powerful and easily accessible models. We need more general methods for obtaining models with practical value at low costs. We need models that can be trained on distributed clusters and achieve relatively high performance without requiring special designs by high-level experts. This can make producing models easier, making AI applications more accessible and allowing more companies to apply deep learning models in business activities.

The deep ensemble model causes many weak learners to integrate into strong learners using bagging, boosting and other methods. Many studies have confirmed that ensemble base learners using various strategies exhibit stronger performances and more robustness than single classifiers^5^. However, most of the existing papers focus on achieving higher test accuracy by designing different ensemble architectures without considering the enormous computation and design costs. These performance-oriented ensembles are not conducive to business applications. On the other hand, the ensemble algorithm of random forest^6^ that constructs multiple decision trees in parallel based on sample and feature sampling can effectively improve the computational efficiency. The classic random forest algorithm has many advantages. Since all the trees are built independently, we can use massive parallelism for computation. Meanwhile, each tree uses only part of the features and part of the samples, which limits the amount of computation required per tree. Moreover, this is a natural distributed computing framework, where each node stores a portion of the data for training its own tree. Finally, we do not need to transfer data when integrating trees into forests, which aligns with the current idea of federal learning.

In this paper, we propose a fragmented neural network ensemble method based on the random forest sampling strategy, i.e., constructing many weak neural networks to eventually ensemble into a strong neural network. Weak neural networks are trained using feature-sampled and sample-sampled data, resulting in weak learners that are detail-oriented and randomized. The fragmented network is constructed for the following reasons:

1. The main purpose of the fragmented network is not to achieve a state-of-the-art model exhibiting high accuracy but rather a model that can be easily implemented and applied under limited computation and human labor conditions.
2. Based on evaluated scenarios, experts do not need to be much involved in the design and optimization of the model architecture, and the base learners are all structurally simple models.
3. Parallel and distributed computation can be easily performed, thus transforming the time efficiency problem into an engineering and control problem.

In this paper, MNIST handwriting classification and recognition data and CIFAR10 classification data are used to build a fragmented neural network for image classification. The goal is to propose a learning mechanism and demonstrate that ensemble neural networks can be used instead of large and complex networks to a certain extent.

In summary, an ensemble learning approach based on a voting system is proposed in this paper. This approach is a model strategy, not a specific model. A strong model is formed by ensemble weak models and achieves the goals mentioned above. Finally, the model effect is validated on the MNIST and CIFAR10 datasets.

## Related work

### Ensemble neural networks

An ensemble neural network trains multiple networks first and then combines the predictions of these models by specific fusion strategies, eventually obtaining higher accuracy than each separate models due to the diversity among the base learners^7^. The ensemble strategy primarily includes bagging, boosting, stacking, homogeneous and heterogeneous ensembles^8^. Among these, bagging is an effective method that can improve the computational speed through parallelism. In this paper, we learn the sampling and ensemble method of random forest to build image classification neural networks.

#### Ensemble strategies

To obtain a more general ensemble neural network that does not require elaborate designs, Alvear-Sandoval et al.^9^ used the SDAE-3 network as the base model and used bagging and class switching to improve the diversity of the ensemble model. The results demonstrated that the model performs well on the MNIST dataset.

Ensemble models can significantly improve accuracy but require considerable time and computational resources, which make them difficult to implement in practical situations. To alleviate this problem, Yang et al.^10^ proposed the FTBME model, which applies three strategies; feature transferring, a random greedy algorithm and fusing weight space. Experimenting on a classic image classification dataset shows that the model can effectively reduce the time cost while maintaining accuracy.

Yoon et al.^11^ proposed a knowledge distillation algorithm that trains a single network with the results of the ensemble model. After segmenting the face into different subregions and training the ensemble network separately, the output probability is trained as the input of a single neural network in the face recognition task. The results show that the accuracy of the ensemble network can be maintained while the time and space costs are reduced.

Cao et al.^12^ proposed an implicit ensemble model that condenses the training results of the ensemble model into a single model. It can effectively reduce the training cost but loses the diversity of the ensemble model because the features captured by each base model at the beginning layers may be very similar.

Katuwal et al.^13^ proposed the edRVFL ensemble neural network. In contrast to traditional ensemble models that train multiple models, they trained only one dRVFL model and then equally treated the results of each hidden layer as the outputs. They obtained excellent results on each benchmark dataset.

#### Decision fusion strategies

The final stage of ensemble learning requires a decision strategy to make the final prediction. In recent years, many in-depth studies have addressed this issue:

Sánchez-Morales et al.^14^ used CNNs, CapsNets and CDAE as base learners, the output probabilities of each model as features, and the K-NN algorithm for training to obtain the weights of each model on each category. Afterward, the categories were weighted to obtain the final category, achieving excellent performance on retinal image data.

Ju et al.^15^ used four decision strategies, unweighted averaging, majority voting, the Bayes optimal classifier, and the super learner, to test the results on different datasets. They found that the unweighted average is significantly better than the other decision strategies when the test accuracies of the base learners are similar. However, the results become unusually sensitive when an overconfident model is present in the base learners; in this case, the super learner performs better.

Xia et al.^16^ proposed a new stacking ensemble model for multiclassification problems. The correlation between the labels is also considered in the stacking process to calculate the weights, and the “accelerated proximal gradient and block coordinate descent optimization” method is then used to accelerate the optimization. This model achieved strong results on a cardiovascular disease dataset.

#### Applications

Due to the good performance and accessibility of deep ensemble models, deep ensemble models are being used to solve problems in an increasing number of fields.

Gifani et al.^17^ trained three neural networks, DenseNet201, ResNet50V2 and Inceptionv3. The models with lower error rates were given higher weights in the decision phase. They obtained 91.62% accuracy on the public covid chest X-ray dataset.

Rai et al.^18^ used CNN and CNN-LSTM for unweighted average ensemble training; moreover, they used the SMOTE-Tomek Link technique to handle imbalanced data, finally obtaining 99.89% accuracy on 123,998 ECG heartbeat samples.

Iqbal et al.^19–21^ trained a CNN that identified diseased cells with 98% accuracy and enabled the discovery of normal and diseased cells.

### Ensemble models on the MNIST dataset

The MNSIT handwritten recognition dataset is divided into a training and a test set, where the training set contains 60,000 images and the test set contains 10,000 images. The MNIST dataset is often used to test the performance of network architectures and to continuously improve the classification accuracy of MNIST datasets. Currently, several state-of-the-art models for handwritten digit recognition using deep ensemble networks exist:

1. Hirata et al.^22^ proposed the EnsNet model, which consists of a CNN and multiple FCSNs. The features of the last convolutional layer in the CNN are divided, and the different subsets of the division are passed as features to the individual FCSNs. Finally, the decision is made using majority voting, obtaining a final accuracy of 99.84% on MNIST.
2. Tabik et al.^23^ proposed a heterogeneous ensemble model, MNIST-net10, where the base model uses several CNN networks with different structures and is ensembled using two different fusion strategies, FS2 and FS3. MNIST-net10 obtained 99.9% accuracy in MNIST.
3. An et al.^24^ trained a CNN model using three different convolutional kernels for two-stage ensembles, i.e., the final model was a heterogeneous ensemble model consisting of three homogeneous ensemble networks. They eventually achieved 99.91% accuracy on MNSIT, the highest accuracy currently achieved on the MNIST dataset.

### Discussions

In most literatures, the main motivations can be summarized quite similar: how to gain a state-of-the-art model, or how to improve the model in some aspects. There are many kinds of model designs due to such a purpose. But the strongest model may not the most useful model. In practical applications, we usually limited by the physical conditions, such as hardware, software, labor and money. It is important to take them into consideration while modeling.

In this paper, the proposed method is carried out under restricted conditions. The parameters and training data cannot be large for each model, which ensures low complexity and low computational effort of each model. All the base models must be independent, which enables parallel and distributed computing. Meanwhile, ensemble method is used to unite all weak models as a way to ensure model effectiveness. These efforts are important for business applications, which means a relatively underpowered model but useful.

## Method

The proposed model is an ensemble neural network with fragmented image data. The training set is first sampled by a certain percentage, while the features are sampled according to different window sizes. n training sets are collected to train n base learners separately and finally generate an ensemble model using various decision strategies. Tabik et al.^23^ showed that increasing the diversity of ensemble models from the perspective of data, models, and decision strategies can effectively improve model generalizability.

**Figure 1:**
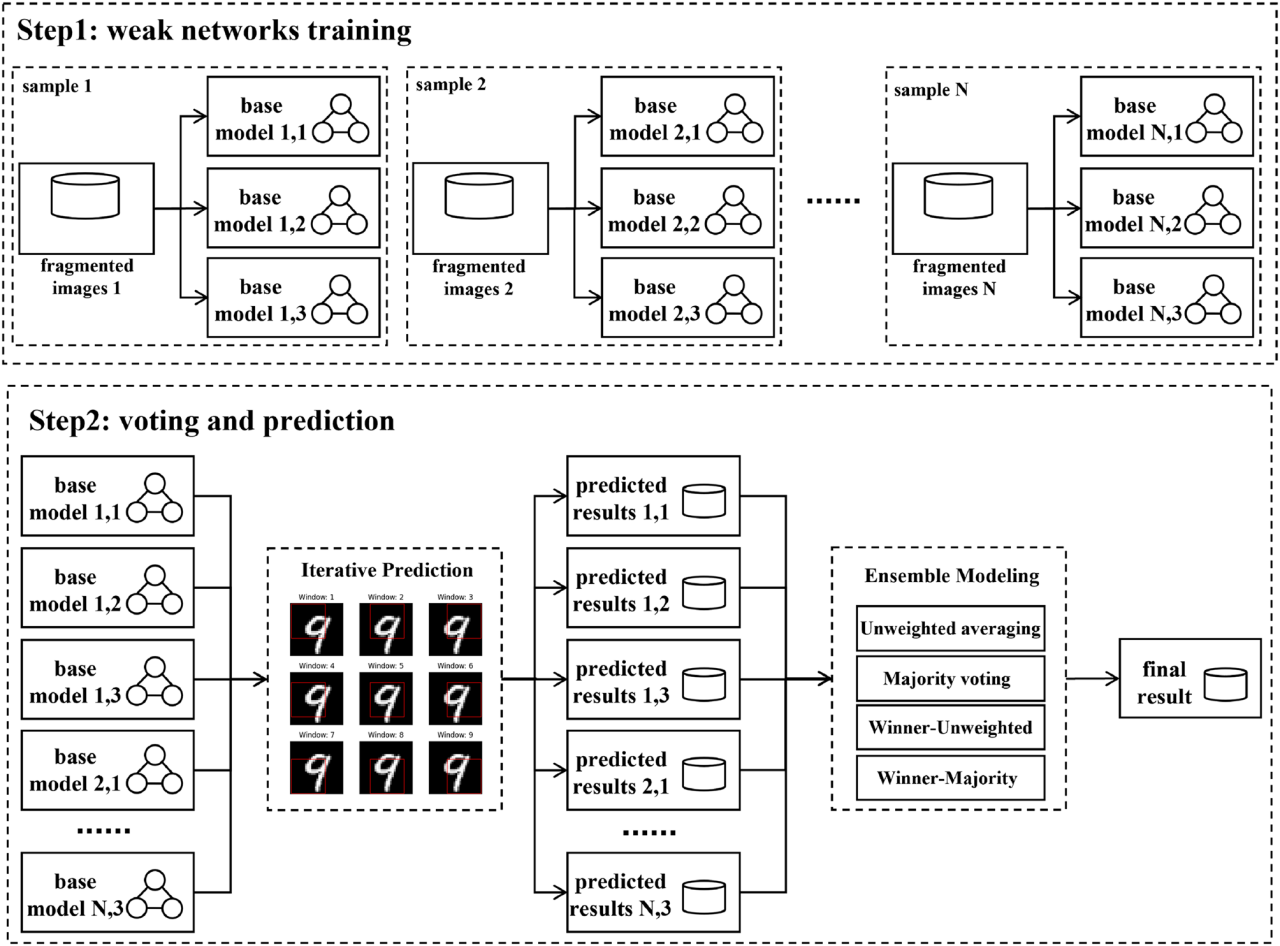
The flow diagram of the proposed method.

For feature sampling, we are inspired by the image processing strategy in the YOLO model^1^. A square or rectangular window is randomly selected as the sampling window for each feature sampling, and only the image fragments within the window are collected. The neighboring pixel information of the image is used, significantly improving the computational efficiency. Three models, the feedforward neural network (FNN), CNN, and deep residual network (ResNet)^25^, are selected as the base learners for constructing the model pool. In the final decision phase, we compare four voting methods of unweighted average and majority voting combining winner-takes-all or not. The fragmented ensemble neural network can effectively reduce the time cost while maintaining an accuracy comparable to that of the full model. The flow diagram of the proposed method is shown in Fig. 1, and the pseudocode of the whole algorithm can be found in Table 1.

**Table 1:** pseudocode of the proposed algorithm.

**Initialize:** window size range $[a,b]$, base models $\{{F}_{i}{\}}_{i=1}^{N}$, train set $X$, test set ${X}_{test}$, number of base learners $N$, voting strategy $V$, sample size $M$ **Output:** ensemble model ${F}_{ens}$, prediction $y$
Step 1: Assign a window size $${C}_{i}$$ to each $${F}_{i}$$. The number of $${F}_{i}$$ assigned per window size is $$N//\left(b-a\right)$$, where window size b is assigned $$N\%\left(b-a\right)$$ base models; Step 2: for each $$1\le i\le N$$ do: $$ {X}_{boot}=sample(X, M)$$ cut images into fragments, $${X}_{boot}=Cut\left({X}_{boot},{C}_{i}\right)$$ fit models, $${F}_{i}={F}_{i}.fit\left({X}_{boot}\right)$$ cut test images into fragments, $${X}_{test}=Cut\left({X}_{test},{C}_{i}\right)$$ predict, $${y}_{i}={F}_{i}.predict\left({X}_{test}\right)$$ Step 3: ensemble, $${F}_{ens}=V\left({\left\{{F}_{i}\right\}}_{i=1}^{N}\right)$$, $$y=V\left({\left\{{y}_{i}\right\}}_{i=1}^{N}\right)$$;
Step 4: return $${F}_{ens}$$, $$y$$

### Consent to participate

Consent to participate was obtained from all participants.

## Experiments

### Data preprocessing

**Figure 2:**
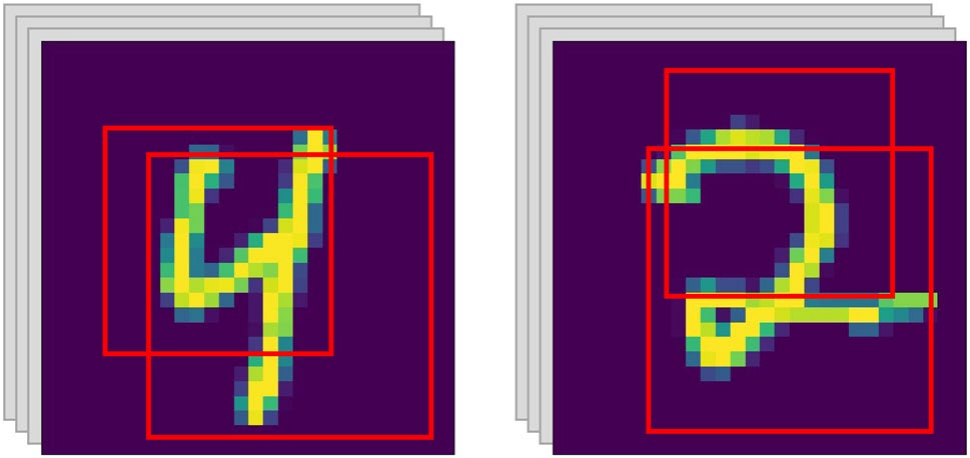
Extracting image fragments on different subsamples with different windows.

The MNIST dataset is divided into training and testing sets, where the training set contains 60,000 images and the testing set contains 10,000 images. Each of the images is a gray handwritten number from 0 to 9 with 28 × 28 pixels and 1 channel. The training and testing sets are first normalized. Then, sampling is conducted in the same way as random forest. First, a certain number of samples are drawn according to the proportion, for example, 10,000 samples with replacement. Afterward, image fragment extraction is performed according to the predefined square window size range $\left[a,b\right]\left(a,b\in N,\le 28\right)$, and the window sizes are equally assigned to each base model. For example, the window size range is^15,19^, and 10 base models are constructed; then, the window sizes of the 10 base models are {15, 15, 16, 16, 17, 17, 18, 18, 19, 19}. Finally, the window location is randomly selected within the image area; only the image fragments of all sampled samples within the window are retained. Figure 2 depicts the schematic diagram of extracting image fragments. For CIFAR10, the data preprocessing is all the same.

### Modeling design

**Figure 3:**
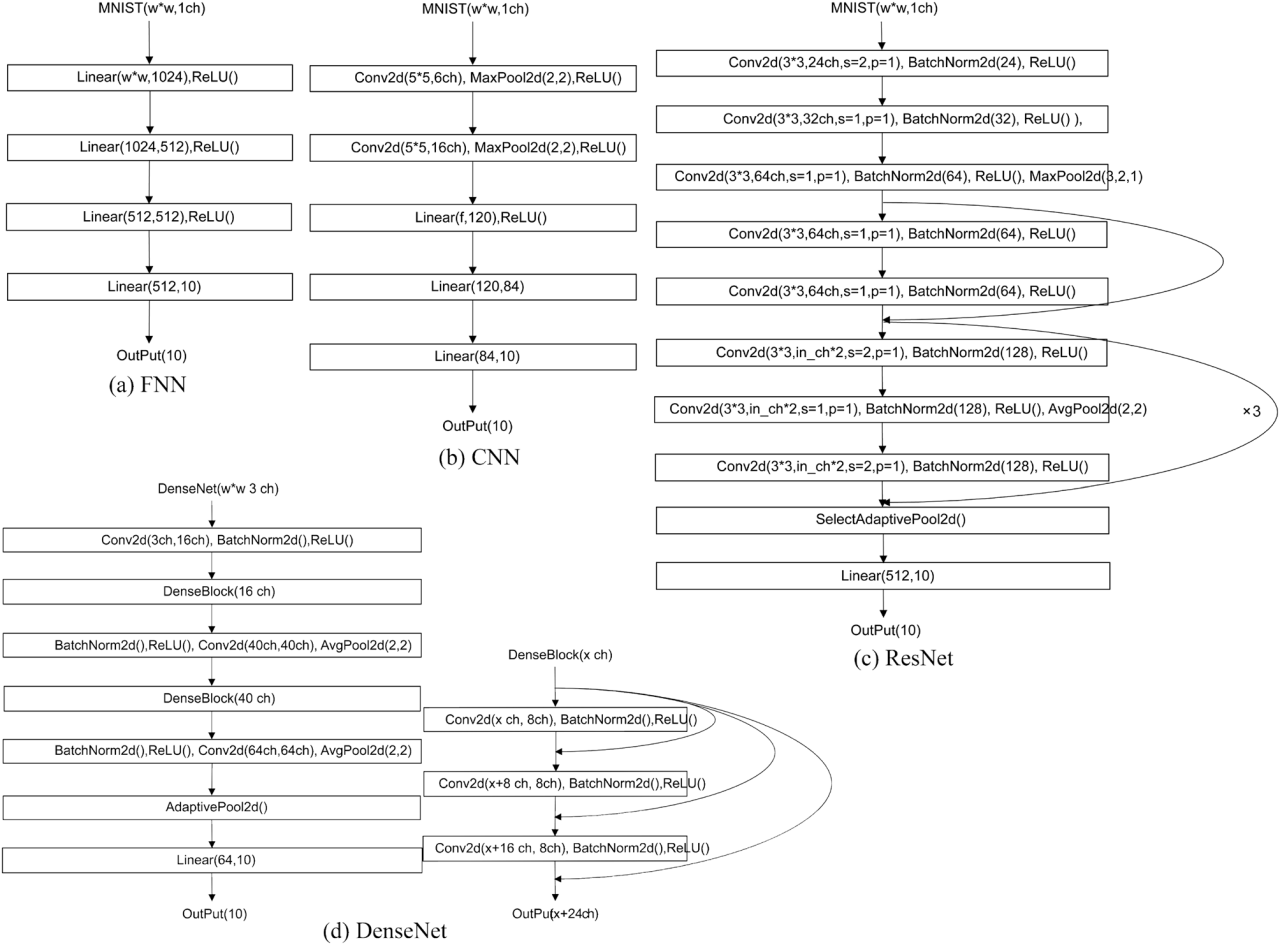
Network architecture of the base models, which are FNN, CNN and ResNet.

FNN, CNN and ResNet networks are selected to construct the model pool for MINIST, and DenseNet for CIFA10. To improve the computational efficiency, the parameters of each model should not be overly complex. For the FNN network, the dataset is first spread as a vector of window size * window length, after which a 4-layer neural network is constructed. The activation function of each layer is the ReLU function, and ten classes are finally output. For the CNN network, 2D convolution is first performed on each convolutional layer, followed by the ReLU activation function and max pooling layer. After the two convolutional layers, three fully connected layers are connected to change the output to ten classes. The ResNet network first performs 2D convolution, after which the BatchNorm layer is connected with the ReLU activation function and a max pooling layer. Afterward, four BasicBlocks are connected, and each BasicBlock contains two layers. Finally, the output is transformed to ten classes using a fully connected network with AdaptiveAvgPool. The underlying architectures of the four neural networks are illustrated in Fig. 3, where $f=16{\left(\frac{\left(\frac{w-4}{2}\right)-4}{2}\right)}^{2}$, *w* represents the size of the input images, and *nch* represents the input number of channels.

After the different base neural networks are selected and trained on fragmented samples, a decision strategy is needed to integrate all the models in the model pool. In this paper, four voting strategies are selected, namely, unweighted voting, majority voting, winner-takes-all-based unweighted voting (winner-unweighted voting) and winner-takes-all-based majority voting (winner-majority voting), where unweighted voting and majority voting are the two commonly used decision strategies in ensemble models^8^. Winner-takes-all indicates that the confidence level is calculated based on the maximum value of each model's predicted probabilities on 10 categories. The larger the maximum probability value is, the higher the confidence of the model in the prediction, and the greater the weight that should be given. Therefore, only the top n models with the highest confidence level are selected for voting:

1 $${w}_{ij}=\left\{\begin{array}{l}1  \quad \, {p}_{ij}\le large{\left({p}_{ik},B\right)}_{k\in \left[1,C\right]}\\ 0    \quad \, else \, \end{array}\right.,$$

where ${w}_{ij}$ represents the weight of the base model $j$ for the sample $i$, ${p}_{ij}$ represents the maximum of the output probabilities of the base model $j$ for the 10 categories, $B$ represents the number of base models to be selected, $C$ is the number of all base models, and $large\left({p}_{ik},B\right)$ represents the head $B$ largest values in ${p}_{ik}$.

Unweighted voting is a soft voting method. The probabilities of all base learners are gathered together, and the average probability values of all classes are output as unweighted averages. Finally, the category with the highest probability is output as the prediction. In contrast, majority voting is a hard voting method. First, he predicted classes are obtained based on the output probabilities of each learner, and the class with the most votes among the predicted classes of all base learners is then output. The unweighted average may be more sensitive to overconfident models.

Finally, the models are evaluated by accuracy, which can be described by formula:

2 $$accuracy=number of correctly classified samples/total sample size.$$

## Results

### Results on MINIST

**Figure 4:**
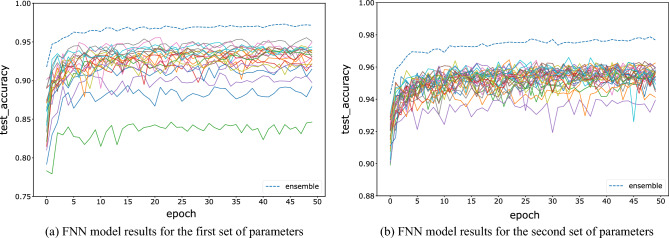
Comparison of the testing accuracy of each base learner of the FNN with the ensemble model during training.

**Figure 5:**
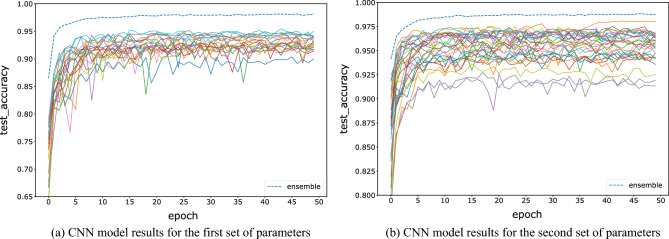
Comparison of the testing accuracy of each CNN base model with the ensemble model during training.

**Figure 6:**
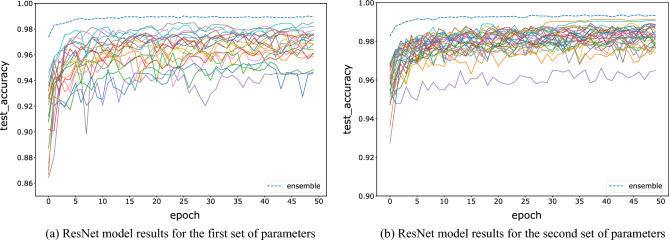
Comparison of the test accuracy of each ResNet base model with the ensemble model during training.

For the FNN, CNN, and ResNet networks, we test two groups of hyperparameters, and each model is trained for 50 epochs. The tested accuracy barely improves after 50 epochs of training. The initial parameters are initialized randomly. The first set of hyperparameters is trained with 20 base learners, and the sample size is 10,000, which reduces the computing complexity while maintaining the model accuracy. The sampling window size is set between 16 and 20, which is equally distributed among the 20 base learners. In this paper, the decision strategy is fixed on the winner-majority vote method, i.e., for each image fragment, the models with the highest predicted probability are selected for majority voting. The number of base learners is increased to 30 for the second set of hyperparameters, and the sample size is increased to 15,000. The sampling window size ranges from 18 to 22, increasing the accuracy of the base models to test the stability of the ensemble results. Figures 4, 5 and 6 show the variations between the accuracies of all base learners and the ensemble model on test data, with increasing epochs under two sets of parameters and three kinds of networks. All results show that the accuracy of the ensemble model is significantly higher than the accuracy of each base learner. This indicates that the advantages of each base learner can be effectively fused by an ensemble to improve accuracy. The bottom solid line indicates that the model tested accuracy is always the lowest, which may be due to the low amount of information contained in that image fragment.

Meanwhile, as shown by the results in Table 2, as the model parameters and complexity of FNN, CNN and ResNet increase, the average accuracy of the base model and the accuracy of the ensemble model increase significantly, which indicates that the accuracy of the ensemble model is highly dependent on the training effect of the base model.

**Table 2:** Comparison of each ensemble model with its base models, where 20 in FNN-20 represents the number of base models.

	FNN-20	FNN-30	CNN-20	CNN-30	ResNet-20	ResNet-30
Maximum accuracy in base models	0.9506	0.9625	0.9497	0.9804	0.9855	0.9912
Ensemble model accuracy	0.9715	0.9768	0.9814	0.9874	0.9895	0.9934

**Figure 7:**
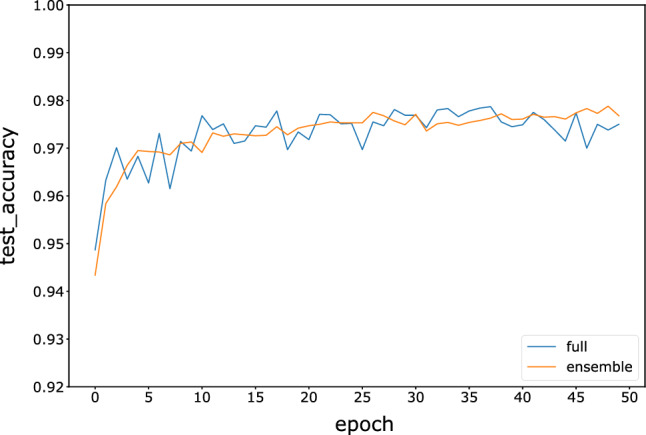
Variation in the test accuracy of the FNN ensemble model and full model during training.

**Figure 8:**
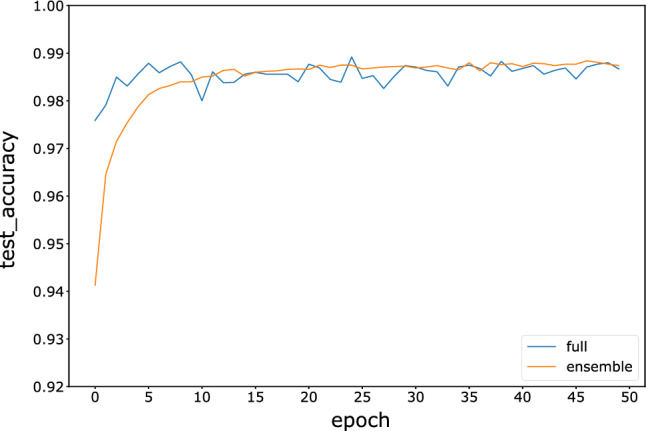
Variation in the test accuracy of the CNN ensemble model and full models during training.

**Figure 9:**
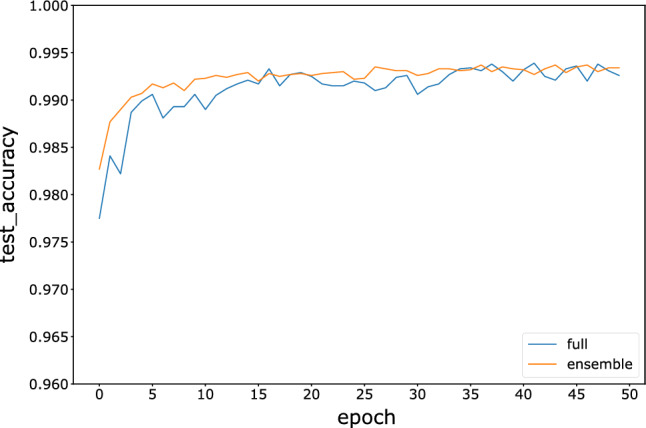
Variation in the test accuracy of the ResNet ensemble model and full model during training.

To compare with the full model, the full model of the three neural networks is trained with 50 epochs on all 60,000 images. The accuracy comparisons between the full models and ensemble models with the winner-majority voting strategy are shown in Figs. 7, 8, and 9. The results indicate that the accuracy of the fragmented ensemble network of the three models is comparable to or even exceeds the accuracy of the full model. Meanwhile, the ensemble model is more stable in terms of test accuracy than the full model, indicating better robustness. This suggests that weak neural network integration using fragmented images can be assumed to be an approximate substitute for a strong neural network. Since weak neural networks are independent of each other and simple in structure, we can easily implement engineering techniques such as parallel and distributed computing to improve computational efficiency.

The accuracy of the ensemble model under the four voting strategies is depicted in Table 3. The results show that unweighted voting has the highest average accuracy. However, unweighted voting requires more time than the other voting strategies since the prediction results of all the base learners are used, which is more obvious when the data volume is large.

**Table 3:** Test accuracy of each ensemble model under the four voting strategies compared to the full model.

	Full model	Winner-unweighted	Majority	Winner-majority	Unweighted
FNN	0.9787	0.9801	0.9812	0.9804	0.9813
CNN	0.9892	0.9884	0.9884	0.9889	0.9885
ResNet	0.9939	0.9937	0.9942	0.9937	0.9941

**Figure 10:**
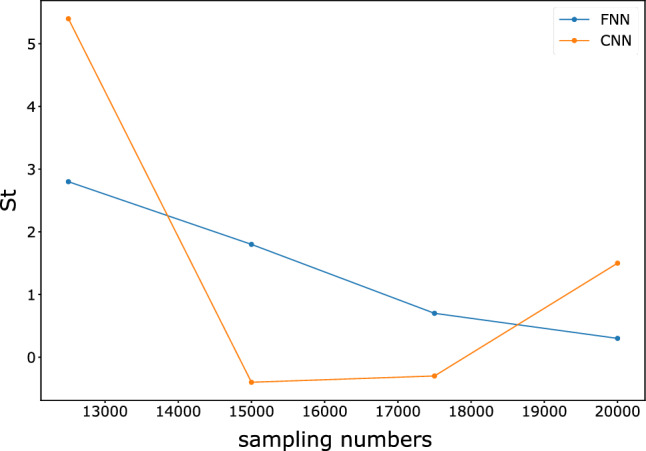
Variation in *St* for FNN and CNN with different sample sizes.

**Figure 11:**
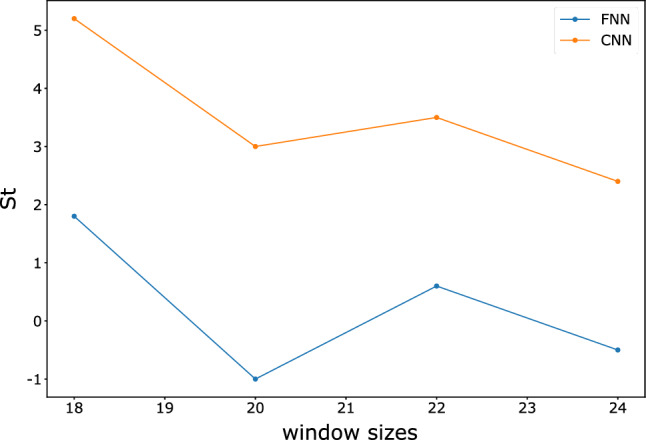
Variation in *St* for the FNN and CNN with different window sizes.

**Figure 12:**
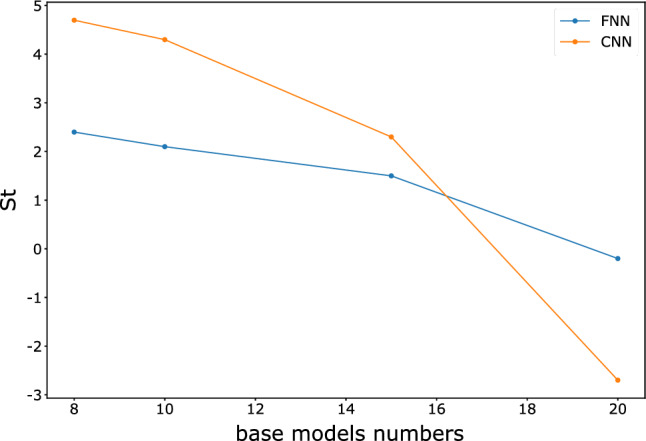
Variation in *St* values for FNN and CNN with different numbers of base learners.

Afterward, we gradually increase the number of base learners, the sample size, and the sampling window size to observe the variations in model accuracy and time consumption. To simplify the calculation, we use 15 training epochs because convergence can almost occur in 15 training epochs. The training is conducted for the FNN and CNN models using the same number of processes as the number of base learners to parallelize the computation. For each set of parameters, we train ten groups of models and calculate the time intervals consumption and accuracy. To compare the gains of the models under parameter variations, we use the following metric as a measure:

3 $$St=\sigma \Delta acc/{e}^{\frac{\Delta t}{\eta }},$$

where $\sigma$ and $\eta$ are the tuning parameters, $\Delta acc$ represents the accuracy variation, and $\Delta t$ represents the time variation. We aim tune the results to a smaller interval to make them more observable, and thus, we set $\sigma =1\times 1{0}^{3}$, $\eta =1\times 1{0}^{5}$. We hope that the changes in parameters will result in a larger accuracy gain with a smaller increase in training time. Figures 10, 11, and 12 show the changes in $St$ as the sample size, sampling window size, and number of models increase.

The results show that the gain from the ensemble model gradually decreases as the sample size increases, which means that the accuracy improvement decreases while the time consumption rapidly increases. The increase in the number of learners greatly benefits the ensemble model in the early stage, but the benefit no longer improves significantly when the number of learners reaches 20. The effects of the sampling window size on the FNN and CNN differ. The variation in the window size of the FNN does not significantly impact the training time, and accuracy does not significantly increase. In contrast, the accuracy of the CNN is more sensitive to the sampling window size due to the convolutional layers in the model. Therefore, a larger sampling window size of the CNN yields a strong improvement in $St$.

Tables 4, 5 and 6 show detailed information about the time consumption, accuracy, and *St* of the FNN and CNN. The complex base learners do not have a significant advantage over the simple base learners under the random forest style ensemble network with voting decisions. Considering time consumption, they may be even less cost-effective.

**Table 4:** FNN and CNN for different sample sizes in terms of time, accuracy and *St.*

Sample size	10,000		12,500		15,000		17,500		20,000	
Model	FNN	CNN	FNN	CNN	FNN	CNN	FNN	CNN	FNN	CNN
Time (s)	156.80 ± 0.172	127.53 ± 0.23	178.25 ± 11.36	146.41 ± 1.81	209.21 ± 4.68	164.71 ± 1.27	234.48 ± 4.41	179.99 ± 1.30	275.29 ± 3.82	197.87 ± 0.53
Accuracy	0.9715 ± 0.0004	0.9740 ± 0.0033	0.9743 ± 0.0001	0.9794 ± 0.0023	0.9761 ± 0.0002	0.9790 ± 0.0021	0.9768 ± 0.0002	0.9787 ± 0.0004	0.9771 ± 0.0009	0.9802 ± 0.0017
*St*	–	–	2.8	5.4	1.8	−0.4	0.7	−0.3	0.3	1.5

**Table 5:** FNN and CNN for different sampling window sizes in terms of time, accuracy and *St.*

Sampling window size		(16, 20)	(18, 22)		(20, 24)		(22, 26)		(24, 28)	
Model	FNN	CNN	FNN	CNN	FNN	CNN	FNN	CNN	FNN	CNN
Time(s)	156.80 ± 0.172	127.53 ± 0.23	154.28 ± 1.98	136.19 ± 0.27	153.33 ± 1.83	142.03 ± 0.66	162.01 ± 4.23	151.28 ± 2.48	163.82 ± 0.21	153.93 ± 0.80
accuracy	0.9715 ± 0.0004	0.9740 ± 0.0033	0.9733 ± 0.0003	0.9792 ± 0.0003	0.9723 ± 0.0002	0.9822 ± 0.0004	0.9729 ± 0.0008	0.9857 ± 0.0002	0.9724 ± 0.0005	0.9881 ± 0.0001
*St*	–	–	1.8	5.2	−1	3	0.6	3.5	−0.5	2.4

**Table 6:** FNN and CNN for different numbers of base learners in terms of time, accuracy and *St.*

Number of base learners		5	8		10		15		20	
Model	FNN	CNN	FNN	CNN	FNN	CNN	FNN	CNN	FNN	CNN
Time (s)	104.28 ± 2.17	83.67 ± 11.93	156.80 ± 0.172	127.53 ± 0.23	206.69 ± 2.11	166.19 ± 13.62	245.97 ± 0.84	207.66 ± 1.54	307.95 ± 0.25	256.50 ± 7.94
Accuracy	0.9691 ± 0.0026	0.9783 ± 0.0008	0.9715 ± 0.0004	0.9740 ± 0.0033	0.9736 ± 0.0014	0.9783 ± 0.0016	0.9751 ± 0.0010	0.9806 ± 0.0004	0.9749 ± 0.0003	0.9779 ± 0.0010
*St*	–	–	2.4	4.7	2.1	4.3	1.5	2.3	−0.2	−2.7

### Results on CIFAR10

**Figure 13:**
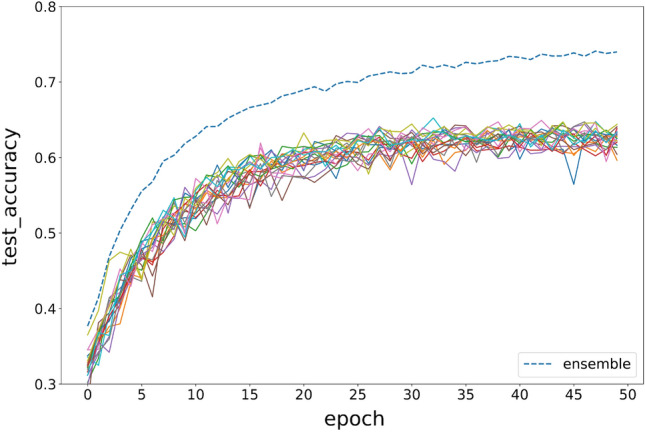
Comparison of the testing accuracy of each DenseNet base model with the ensemble model during training.

DenseNet is used to build model pool for CIFAR10, since the previous research shows it is better for high noisy data like CIFAR10. The modeling process is the same with MINIST. 20 base learners are trained with 50 epochs, the sample size is 10,000, the sampling window size is set between 25 and 29. The test accuracy of ensemble model and base models are shown in Fig. 13. The results are the same with MINIST, ensemble model is significant higher than any base model. Ensemble of week models with fragmented images is effective.

**Figure 14:**
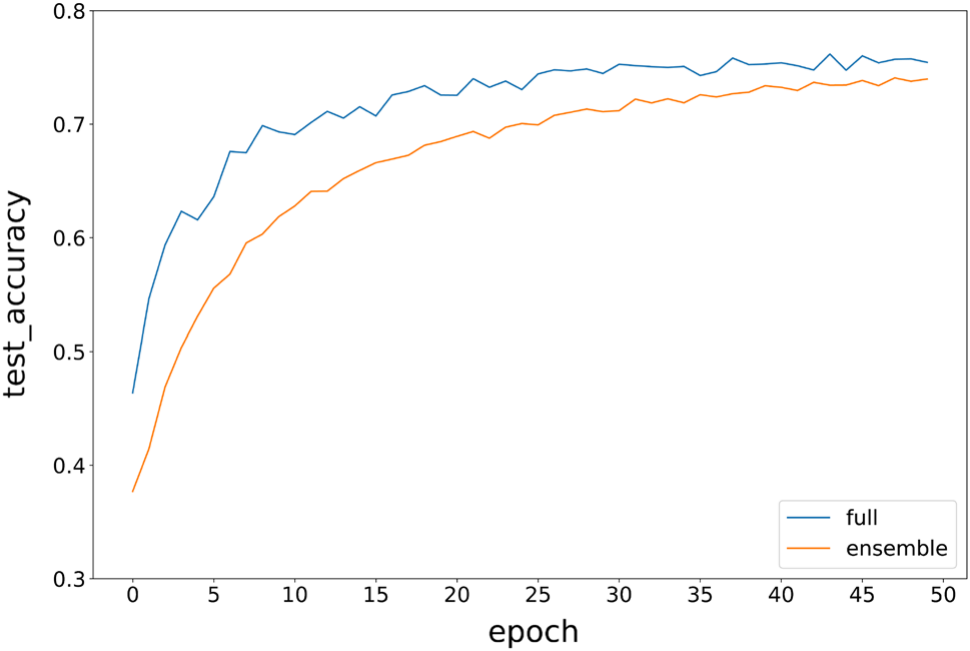
Variation in the test accuracy of the DenseNet ensemble model and full models during training.

Figure 14 is the comparation of ensemble model with full model. We found it is not that good like MINIST, since the accuracy of ensemble model is 0.7407 and does not reach that of full model of 0.7617. That is because CIFAR10 contains much more noise than MINIST, the full model with larger parameters and full image will gain more advantages than MINIST. However, the accuracy of ensemble model still converges on full model after 50 epochs. The ensemble method still reaches our expectation, it is a useful method.

### Heterogeneous model pool

A heterogeneous model pool means that each base learner should be as diverse as possible in terms of samples, features, and model structures while maintaining a certain accuracy. This allows the final model to combine different learning preferences of each base learner to obtain a stronger learning capability. To compare the heterogeneous ensemble model with the homogeneous ensemble model, we select the FNN, CNN, support vector machine (SVM), eXtreme Gradient Boosting (XGBoost), and ResNet10t models for training. First, the sampling window size ranges between^16,22^, which is equally distributed to each model, i.e., each model trains 5 base models with different sampling window sizes. Table 7 depicts the highest tested accuracy of each ensemble model under the four voting strategies. The ensemble of all five kinds of base learners yields an accuracy of 98.15%. Although this significantly higher than four of the homogeneous ensemble models, it is lower than the 99.11% accuracy of the ResNet ensemble. When ResNet is removed, the accuracy of the ensemble network reaches 97.55%, which is higher than the accuracy of the four ensemble models. Ensemble-5 achieves a lower accuracy than ResNet because unweighted voting is very sensitive to overconfident base learners. The base learners must be somewhat comparable when using average voting. Additionally, structural differences or different learning preferences among the learners are required for better integration. Utilizing a super learner may be a good strategy when an overconfident base learner is used^15^. However, the proposed approach uses bootstrap sampling and does not apply cross-validation in optimization, so it is not suitable for use with a super learner.

**Table 7:** Comparison of the test accuracy of each ensemble model with heterogeneous ensemble models.

Model	XGBoost ensemble	SVM ensemble	FNN ensemble	CNN ensemble	ResNet ensemble	Ensemble-4	Ensemble-5
Accuracy	0.9627	0.9703	0.97	0.9748	0.9911	0.9755	0.9815

ensemble-4 and ensemble-5 are heterogeneous ensemble models with all kinds of base learners, where ensemble-4 represents the ensemble of all models except the ResNet networks, and ensemble-5 represents the ensemble of all models.

## Conclusions

In this paper, we propose a fragmented neural network approach that ensembles many small weak networks. This approach is expected to reduce the technical difficulty and hardware requirements of deep learning and thus provide an AI approach that is more accessible for practical use. We conduct experiments on the MNIST and CIFAR10 datasets with FNN, CNN, DenseNet, and ResNet as the base network structure. The following conclusions are obtained by comparing the ensemble weak networks and the single full model.

1. By using fragmented images to build weak neural networks as base learners, the accuracy of the ensemble model is significantly higher than that of each base learner. Although each base learner can represent only the information of its randomly extracted image fragments, the ensemble network can still effectively fuse the information of each base learner. Thus, similar to random forest, a strong ensemble neural network with higher accuracy is created. Meanwhile, the accuracy of the ensemble network increases significantly as the base learner becomes more complex; that is, the accuracy of the ensemble network is highly dependent on the performance of the base learners.
2. Comparing the ensemble network with the full neural network, which is trained with full data shows that the accuracy of the ensemble network is comparable to or even exceeds the accuracy of the full network. The accuracy of the ensemble network is also more stable than that of the full model, which suggests that the ensemble network can achieve better robustness. It is reasonable to conclude that the ensemble of weak networks trained with fragmented images can be an approximate substitute for the full network. In addition, since the weak networks are independent of each other and have a simple structure, we can use parallel and distributed computing techniques to reduce the time consumption.
3. We can build a heterogeneous model pool, which allows the ensemble model to have multiple learning preferences. This can effectively enhance model diversity and improve model performance.

## Future work

The research in this paper still has many shortcomings, for example, the way of model ensemble is relatively simple and the model pool is not diversified enough.

In future work, we plan to conduct further research on fragmented ensemble neural networks. We hope to build a control system for dynamic model pools that can easily conduct operations such as adding, deleting, and querying to the model pool in engineering applications. This allows us to inspect the performance, samples, and features of each base learner in real time. We can also eliminate poor models or outdated models and then integrate new models trained with new data in real time, which provides evolutionary capability to the ensemble model.

On the other hand, since the base learners exhibit good independence from each other, we can try to apply this method in federated learning. Each data node can be considered as a fragment of data. We can train the model locally at each data node and then transfer the models to the central node. Ensemble decisions are formed at the central node through voting. This is simpler than transmitting parameters for federated learning, and it significantly reduces the communication cost.

## Data Availability

The datasets used in this work come from public datasets, which are available at http://yann.lecun.com/exdb/mnist/, and https://www.cs.toronto.edu/~kriz/cifar.html.
